# Impact of preweaning vaccination on host gene expression and antibody titers in healthy beef calves

**DOI:** 10.3389/fvets.2022.1010039

**Published:** 2022-09-26

**Authors:** Matthew A. Scott, Amelia R. Woolums, Brandi B. Karisch, Kelsey M. Harvey, Sarah F. Capik

**Affiliations:** ^1^Veterinary Education, Research, and Outreach Center, Texas A&M University and West Texas A&M University, Canyon, TX, United States; ^2^Department of Pathobiology and Population Medicine, Mississippi State University, Mississippi State, MS, United States; ^3^Department of Animal and Dairy Sciences, Mississippi State University, Mississippi State, MS, United States; ^4^Prairie Research Unit, Mississippi State University, Prairie, MS, United States; ^5^Texas A&M AgriLife Research, Texas A&M University System, Amarillo, TX, United States; ^6^Department of Veterinary Pathobiology, School of Veterinary Medicine and Biomedical Sciences, Texas A&M University, College Station, TX, United States

**Keywords:** beef calves, vaccination, preweaned calves, bovine respiratory disease, immunity, RNA sequencing (RNA-Seq), T-cell, inflammation

## Abstract

The impact of preweaning vaccination for bovine respiratory viruses on cattle health and subsequent bovine respiratory disease morbidity has been widely studied yet questions remain regarding the impact of these vaccines on host response and gene expression. Six randomly selected calves were vaccinated twice preweaning (T1 and T3) with a modified live vaccine for respiratory pathogens and 6 randomly selected calves were left unvaccinated. Whole blood samples were taken at first vaccination (T1), seven days later (T2), at revaccination and castration (T3), and at weaning (T4), and utilized for RNA isolation and sequencing. Serum from T3 and T4 was analyzed for antibodies to BRSV, BVDV1a, and BHV1. Sequenced RNA for all 48 samples was bioinformatically processed with a HISAT2/StringTie pipeline, utilizing reference guided assembly with the ARS-UCD1.2 bovine genome. Differentially expressed genes were identified through analyzing the impact of time across all calves, influence of vaccination across treatment groups at each timepoint, and the interaction of time and vaccination. Calves, regardless of vaccine administration, demonstrated an increase in gene expression over time related to specialized proresolving mediator production, lipid metabolism, and stimulation of immunoregulatory T-cells. Vaccination was associated with gene expression related to natural killer cell activity and helper T-cell differentiation, enriching for an upregulation in Th17-related gene expression, and downregulated genes involved in complement system activity and coagulation mechanisms. Type-1 interferon production was unaffected by the influence of vaccination nor time. To our knowledge, this is the first study to evaluate mechanisms of vaccination and development in healthy calves through RNA sequencing analysis.

## Introduction

Vaccination remains one of the most important tools for controlling bovine respiratory disease (BRD) in beef calves (1). Increasing adaptive immunity against known pathogens is the goal of vaccination; however, the presentation of antigens to the immune system elicits multiple cascading events within an animal as part of both innate and adaptive immunity. The most commonly measured indicators of adaptive immunity are serum antibody titers to specific pathogens of interest. Although useful, antibody titers have some limitations including multiple samples must be taken for accurate diagnosis of infection by endemic respiratory agents, and sufficient time must pass for the immune system to respond adequately (2, 3). One alternative to antibody titers is to evaluate differential gene expression to identify markers that indicate immune competency, immune responsiveness, and/or predict future immunity to those pathogens. However, knowledge gaps remain regarding the impact of vaccination on gene expression and how that gene expression may correlate with the development of adaptive immunity.

Although commercially available vaccines have been evaluated and approved by the USDA APHIS Center for Veterinary Biologics for purity, safety, potency, and efficacy, the requirements for efficacy studies are often quite different than the conditions under which the vaccine will be used in the field. While challenge studies can be very useful tools (4), they do not accurately model natural bovine respiratory disease and are often done with seronegative calves that have not been exposed to any pathogens or stressors. Strict protocols and timing of administration are also followed. In contrast, beef producers often use vaccines at different intervals from the label, in animals with a variety of backgrounds and nutritional, immune function, or passive transfer status, and often in the face of bacterial or viral exposure or other stressors (5). These differences can make it difficult to achieve the efficacy seen in the tightly controlled approval studies and raises the question whether vaccines, as commercially employed, are influencing rates of morbidity and performance in a consistent manner. To answer this question, the cattle industry needs additional research on these vaccines as they are employed in natural field conditions and the impact they have on cattle health, performance, and immune function.

Given this background, our objective was to explore differences in host gene expression in calves that were vaccinated preweaning with a modified live vaccine for respiratory pathogens or not *via* time-course transcriptomics, and to pair those data with antibody titers and health records. These data will support exploration of associations and generation of hypotheses regarding the immune response to vaccination that may influence future research and use of vaccines in preweaned beef calves.

## Materials and methods

### Animal use and study enrollment

All animal use and procedures were approved by the Mississippi State University Animal Care and Use Committee (IACUC protocol #19-169) and carried out in accordance with relevant IACUC and agency guidelines and regulations. This study was carried out in accordance with Animal Research: Reporting of *In Vivo* Experiments (ARRIVE) guidelines (6).

Eighty-four bull calves were enrolled in a split plot design study to evaluate the impact of different management strategies on BRD morbidity, mortality, and performance (7). Animals were randomly assigned to whole plot (VAX or NOVAX) which were housed in 6 pastures during the cow-calf phase with no nose-to-nose contact. They were also randomly assigned to split plot level treatment of being directly transported to Texas for backgrounding after weaning (DIRECT) or sent to an auction market and then an order buyer facility for 3 days prior to transport to Texas for backgrounding (AUCTION); this event occurred after the timepoint T4, described below. All animals were visually assessed each day for signs of BRD and/or other disease by trained university employees and detailed health histories were kept on each calf. The observed signs of BRD were assigned a severity score of 0–4, adapted from the scoring system previously described by Holland et al. (8).

Calves were evaluated at four time points, described as T1, T2, T3, and T4. At T1, calves were vaccinated with 2 ml Pyramid 5 (Boehringer Ingelheim Animal Health) subcutaneously (VAX) or given 2 ml 0.9% saline subcutaneously (NOVAX) (median age = 107 days). Additionally, calves were tested *via* ear notch ELISA to evaluate PI status at T1; no PI positive calves were found. At T2, or 7 days post-vaccination (median age = 114 days), all calves were weighed and sampled. At T3 VAX calves were again administered (revaccinated with) 2 ml Pyramid 5 subcutaneously and NOVAX calves were given 2 ml 0.9% saline subcutaneously (median age = 183 days); all calves were castrated by knife with no analgesia on T3. All calves also received 5 ml of a multivalent clostridial bacterin-toxoid (Covexin 8, Merck Animal Health) subcutaneously at time point T1 and T3). All calves were handled so that no contact between vaccinated and non-vaccinated calves would occur. Calves were abruptly weaned at T4 (median age = 230 days) and entered the next phase of the study where they were kept in their original pastures in Mississippi for 3 days before being transported directly from Mississippi to Texas for backgrounding (DIRECT) or sent to an auction market where they stayed in a pen not in contact with other cattle for approximately 6 h, and then were transported for housing at an order buyer facility for 3 days prior to transport to Texas (AUCTION) Non-study calves from other sources were housed at the order buyer facility at the same time as the study calves, but they were not mixed with the study calves. In Texas (samples not evaluated in this study), calves were kept in one of 12 pens corresponding to their original random assignment to whole and split plot treatments (*n* = 3 pens of each pair of treatments). Whole blood was collected from all calves into Tempus RNA blood tubes (Applied Biosystems) and into serum tubes *via* jugular venipuncture immediately prior to first vaccination (T1), seven days post-vaccination (T2), immediately prior to vaccine booster administration and castration (T3), and at time of abrupt weaning (T4; 47 days post-booster). Overall, there were 7 days between T1 and T2, 70 days between T2 and T3, and 47 days between T3 and T4.

Twelve calves that remained clinically unaffected by BRD during the cow-calf and backgrounding phases of production were selected for RNA sequencing *via* stratified random sampling within the calves that remained healthy throughout the study within each backgrounding pen which resulted in 1 calf selected per backgrounding pen (*n* = 3 VAX/DIRECT, *n* = 3 NOVAX/DIRECT, *n* = 3 VAX/AUCTION, and *n* = 3 NOVAX/AUCTION). A total of 48 blood samples across the four time points were analyzed for whole blood transcriptomes. Metadata for all selected calves are found in Supplementary material 1.

### Antibody titers

Serum collected at T3 and T4 was stored at −20°F before analysis at the University of Georgia's Athens Veterinary Diagnostic Laboratory. Serum neutralizing antibodies were assayed for bovine herpesvirus−1 (BHV-1), bovine viral diarrhea virus type 1a (BVDV1a), bovine respiratory syncytial virus (BRSV), and parainfluenza-3 virus (PI-3) per SOP # Ser013. Resulting titer levels for these antibodies are found in Supplementary material 1 and is limited to descriptive analysis only due to the small number of calves (*n* = 12).

### Average daily gain

Differences in average daily gain between T1 and T4 were evaluated *via* generalized linear mixed effect models estimated *via* restricted pseudolikelihood with the Kenward-Rodgers adjustment for degrees of freedom in SAS 9.4. The model included vaccination status as a fixed effect and a random intercept for backgrounding pastures. Differences in least square means are reported and a cutoff of *p* ≤ 0.05 was used to determine significance.

### Next-generation RNA sequencing and bioinformatic data processing

Total RNA isolation, quality control, sequencing library preparation, and sequencing was performed by the Texas A&M University Institute for Genome Sciences and Society (TIGSS; College Station, TX, USA). Total RNA was isolated with Tempus Spin RNA Isolation Kit (Applied Biosystems), based on manufacturer's instructions. Total RNA from each sample was analyzed for RNA concentration and integrity with a Qubit 2.0 Fluorometer (ThermoFisher) and an Agilent 2,200 Bioanalyzer (Agilent), respectively; all RNA samples were of high quality (RIN: 7.8–9.5; mean = 8.8, s.d. = 0.3) and concentrations (ng/μL: 84.1–380.0; mean = 222.4, s.d. = 71.4). Library preparation for mRNA was performed with the TruSeq Stranded mRNA Library Prep Kit (Illumina), following manufacturer's instruction. Paired-end sequencing for 150 base pair read fragments was performed on an Illumina NovaSeq 6000 analyzer (v1.7+; S4 reagent kit v1.5) in one flow cell lane.

Quality assessment of reads was performed with FastQC v0.11.9^1^ and MultiQC v1.12 (9), and read pair trimming for unambiguous base calls, adaptors, and retained minimum read length of 28 bases was performed with Trimmomatic v0.39 (10). Trimmed reads were mapped and indexed to the bovine reference genome assembly ARS-UCD1.2 with HISAT2 v2.2.1 (11). Sequence Alignment/Map (SAM) files were converted to Binary Alignment Map (BAM) files, prior to transcript assembly, with Samtools v1.14 (12). Transcript assembly and gene-level expression estimation for differential expression analysis was performed with StringTie v2.1.7 (13), as described by Pertea et al. (14). All sequencing data produced in this study are available at the National Center for Biotechnology Information Gene Expression Omnibus (NCBI-GEO), under the accession number GSE205004.

### Differential gene expression analysis

Gene-level count matrices were processed and analyzed in RStudio, using R v4.1.2. Samples were classified by vaccination group and time point, where raw gene counts were processed and filtered by procedures described by Chen et al. (15). Any gene with a minimum total count above 100 and a count-per-million (CPM) of 0.2 in at least twelve samples was retained for further analysis. Post filtering, the complete dataset was considered non-sparse, and therefore normalized for differential expression analysis with the trimmed mean of M-values method (TMM) (16). Tagwise dispersion estimates of gene counts were supplied into the Bioconductor package glmmSeq v0.1.0^2^ for negative binomial mixed effect modeling of gene counts. The following linear mixed-effect model was fitted to account for time points and vaccination group as fixed effects, and housing (pasture) and individual ID as random effects:


$$\begin{array}{l} Model:\sim Timepoint\;*\;Vaccine\;*\;Timepoint:Vaccine\\ \;+\left(1|Pasture\right)+\left(1|ID\right) \end{array}$$

Model adaptation allowed for the assessment of differentially expressed genes (DEGs) across timepoints, vaccine groups, and the interactions between timepoints and vaccine group, where *p*-values were adjusted for false discovery rates (FDR) with the Benjamini-Hochberg method; genes were considered significantly expressed with an FDR ≥ 0.05. Pairwise comparisons for DEGs between each vaccination groups at every time point and within each vaccination group across each time point was performed with edgeR v3.36.0 (15, 17), fitting genes under generalized linear model (GLM) framework and employing quasi-likelihood F-tests (QLF); pairwise gene comparisons were considered significant with an FDR ≥ 0.10.

### Dimensional reduction and unsupervised clustering analyses

Heatmap, principal component, and clustering analyses were performed with all filtered and log2 count-per-million (log2CPM) values of TMM-normalized gene counts between all 48 samples. Heatmap and exploratory clustering analysis of samples, with respect to vaccination, time points, and individual IDs, were performed with the Bioconductor package pheatmap v1.0.12,^3^ utilizing Canberra distances and Pearson correlation coefficients for unsupervised hierarchical clustering of samples and DEGs, respectively. Specifically, z-scores were calculated and utilized for heatmap analysis from log2CPM values of normalized (TMM) expression values. Gene expression was grouped into 48 distinct clusters with the k-means algorithm embedded within pheatmap; the number of clusters was determined from the Elbow method. High dimensional data exploration and reduction *via* principal component analysis (PCA) was conducted with the Bioconductor package PCAtools v2.0.0,^4^ utilizing a correlation matrix; normalized gene counts were processed through mean centering and variance scaling. A scree plot was generated to determine the number of principal components (PCs) to retain for analysis, utilizing Elbow and Horn's parallel analysis methods (18). A Spearman's rank correlation matrix of retained PCs was constructed with metadata components from all samples, which included individual identification (ID), birthweight, age of animal for each sample (Age), housing pen at Mississippi (Pasture), vaccination group (Vaccine), sampling time point for each sample (Timepoint), and the slope of weight gain over time starting at birth (i.e., growth rate; GR); correlations were considered significant with an FDR ≥ 0.10. To determine genes which were driving the variation seen among each significantly correlated PC, a loadings plot was generated with the top/bottom 2% retained variables across each component loading range. A PCA biplot was constructed from the PCs with significant correlation to vaccination groups; data ellipses were calculated from multivariate t-distributions and encompassed 80% confidence levels of expressional t-distribution across each time point.

### Functional enrichment analyses of DEGs

Differentially expressed genes were analyzed for functional enrichment of gene ontology (GO) terms, Reactome pathways, and KEGG pathways with KOBAS-i (19) (accessed May 2, 2022), utilizing hypergeometric testing and Benjamini-Hockberg adjusted *p*-values (FDR ≥ 0.05). Functional enrichment of DEGs were analyzed in three separate analyses: (1) DEGs shared between time points in both glmmSeq and QLF testing of vaccinated and non-vaccinated calves (i.e., shared genes between glmmSeq–timepoints, QLF Vax T1vsT2, and QLF Novax T1vsT2), (2) DEGs identified between vaccination groups across each time point by both glmmSeq–vaccination and QLF testing (i.e., glmmSeq–Vaccine and Vax vs. Novax at T1), removing DEGs identified by method #1, and (3) DEGs solely identified in glmmSeq analysis of Timepoint: Vaccine interactions; this approach allowed for the independent assessment of functional enrichment influenced by calf development (i.e., time) and vaccine administration. Enriched GO terms and pathways were evaluated for directionality (increased or decreased) based on log2 fold changes of associated DEGs. Clustering and visualization of enriched KEGG terms was performed with the embedded enrichment visualization tool within KOBAS-i, utilizing edge (correlation) thresholds of 0.40 and top *n* clusters set to 8; more information regarding the embedded enrichment visualization tool framework is provided by Bu et al. (19).

## Results

### Antibody titers and average daily gain

Comparison of antibody titers indicated calves were likely naturally infected with BRSV and PI-3, because antibody titers to these agents increased between T3 and T4 in both vaccinated and non-vaccinated calves. Given the small number of calves evaluated, this somewhat clouds our ability to detect the effect of vaccination using serology of samples collected at only two timepoints. However, vaccinated calves appeared to respond well to MLV vaccination as indicated by the BVDV1a titer response (Supplementary material 1). Average daily gain between T1 and T4 was not significantly different (*p* = 0.31) between the VAX (model-adjusted least square mean 1.89 lbs) and NOVAX (model-adjusted least square mean 2.13 lbs) groups.

### Differential gene expression patterns and enriched biological mechanisms

**Figure 1:**
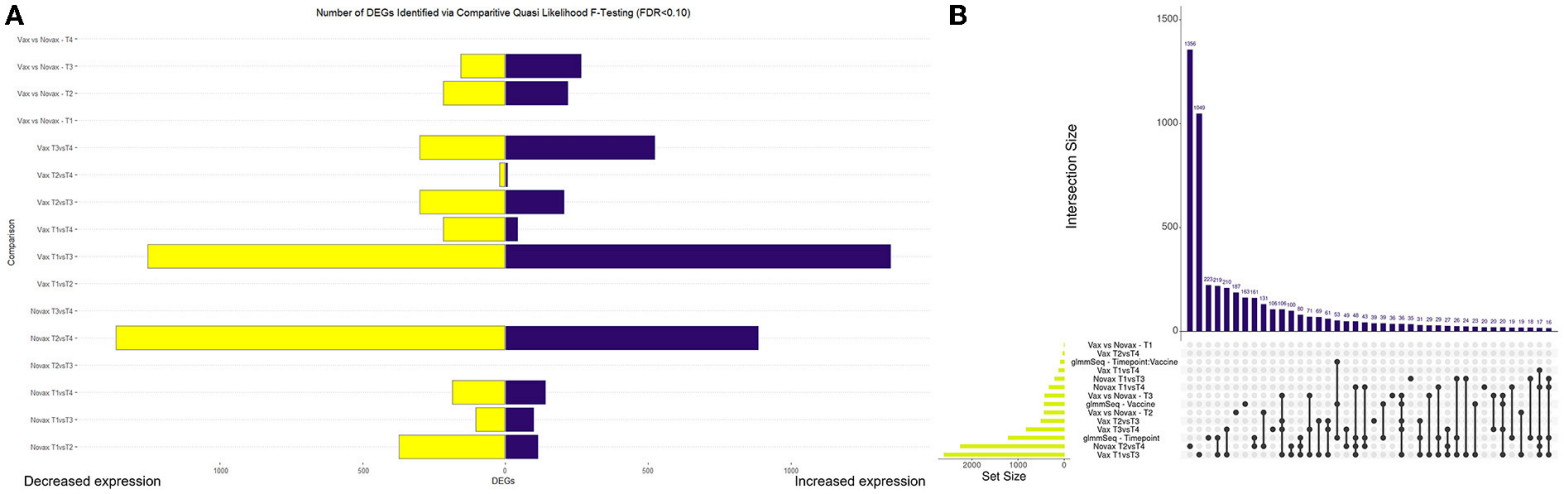
Visualization of differentially expressed genes (DEGs) identified through edgeR Quasi Likelihood F-testing and glmmSeq analyses. **(A)** Bar graph depicting the number and directionality of DEGs found in each edgeR pairwise test. Directionality is based on the first testing group within each pairwise test. For example, Vax T3vsT4 depicts 524 DEGs upregulated and 302 DEGs downregulated at T3 when compared to T4. **(B)** Upset plot demonstrating the number of DEGs overlapped between all differential expression analyses. Novax T2vsT4 possessed the most (1356) unique DEGs of any analysis, while Vax T1vsT3 and glmmSeq–Timepoint possessed the highest number of genes identified in multiple analyses (219).

Read mapping and alignment of the 48 transcriptomes to the ARS-UCD1.2 bovine reference genome resulted in an overall mapping rate average of 95.50% (s.d. = 0.96%). In total, gene-level alignment resulted in a total of 33,310 unique features, with a median library size of 41,089,614 (s.d. = 4,111,721) (Supplementary Figure 1). Pre-processing and filtering of low expression values resulted in a total of 17,371 genes used for downstream analyses (Supplementary Table 2). Analysis of genes from glmmSeq resulted in 1213, 435, and 85 DEGs when evaluating time, vaccination, and the interaction of time and vaccination, respectively (Supplementary Table 3). Comparative analyses for DEGs between vaccination groups and time points was conducted with edgeR GLM-QLF testing. Analysis of the NOVAX group over time yielded a total of 3,271 DEGs across six comparisons (Supplementary material 4). Analysis of the VAX group over time yielded a total of 4,085 DEGs across six comparisons (Supplementary material 5). Analysis of each time point between the VAX and NOVAX groups yielded a total of 861 DEGs across four comparisons (Supplementary material 6). Visualization of the number and directionality of DEGs identified from GLM-QLF testing and overlapping of DEGs from glmmSeq and edgeR QLF analyses, are found in Figure 1.

**Figure 2:**
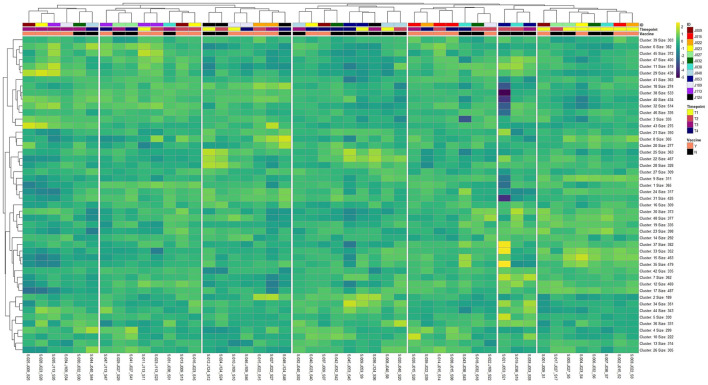
Heatmap and unsupervised hierarchical clustering analysis of global gene expression patterns across all 48 sample libraries (*n* = 17,371) following optimal k-means clustering of genes (k = 48). Gene clusters were labeled by clustering order (Cluster) and the total number of genes embedded within each cluster (Size). Sample libraries were labeled top-to-bottom with individual identification (ID), time point for each sample (Timepoint; T1, T2, T3, and T4), and vaccination group (Vaccine; Yes or No).

Heatmap and unsupervised clustering analysis, seen in Figure 2, demonstrated that the majority of calves (*n* = 7) were highly similar in global gene expression prior to vaccine administration (T1; right side). Time of sampling (Timepoint) emerged as a considerable factor in determining distinction between groups (Vaccine) and individual calves (ID), as the majority of samples on the left side of the heatmap (i.e., furthest from the T1 samples) were at time of vaccine boostering (T3) and weaning (T4). Several individuals (J015, J022, J027, J053, J109, J113, J124) demonstrated high self-similarity in global gene expression between time points.

**Figure 3:**
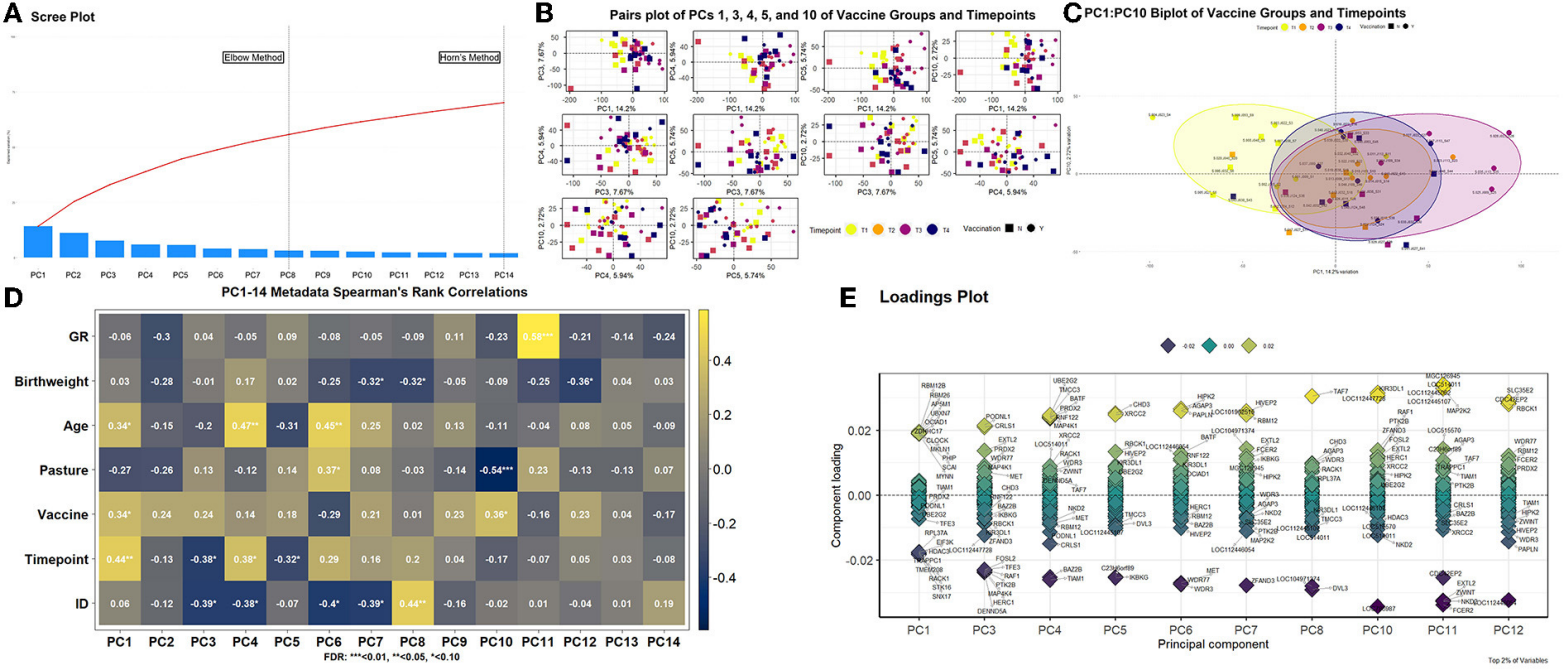
Principal component analysis of global gene expression patterns for all samples. **(A)** Scree plot[[Inline Image]] analysis depicting the maximum number of components to retain. Horn's parallel analysis method was ultimately utilized to retain the first 14 principal components (PCs), which explained 70.15% of total variance across the dataset. **(B)** Multiple biplot analysis (pairs plot) of PCs possessing significant correlation with timepoint and/or vaccination. Each point (vector) represents a PC score of an individual sample, in which the further from plot-center the point is, the more variation that sample contributes to the total variation. The colors yellow, orange, violet, and blue represent the timepoints T1, T2, T3, and T4, respectively; the shapes square or circle represent the vaccination status as no or yes, respectively. **(C)** Specific multivariate biplot analysis of PC1 and PC10, as influenced by timepoint and vaccination (see 3B color and shape coding). **(D)** Spearman's Rank correlation matrix heatmap of retained PCs and corresponding metadata components. Metadata components included the slope of weight gain over time (i.e., growth rate; GR), weight at birth (Birthweight), age at sampling (Age), pasture assignment (Pasture), vaccination status (Vaccine), time of sampling (Timepoint), and individual identification (ID). **(E)** Loading plot analysis with associated genes driving the variation explained by PCs with a significant correlation identified by 3D. Only the top 2% of genes based on component loading scores (i.e., most responsible for explained variation) were retained for each PC.

Multidimensionality analysis and visualization of global gene expression patterns *via* PCA is found in Figure 3. Utilizing both the elbow method and Horn's parallel analysis, a total of 14 principal components (PCs) were determining as optimal for demonstrating explained variation across the 48 transcriptomes; the first 14 PCs retained 70.15% of the variance within the data (Figure 3A). Pairwise plotting of selective PCs (Figures 3B,C) was performed with those PCs which demonstrated significant correlations with timepoints and/or vaccination status (Figure 3D). The first PC, accounting for 14.20% of the total explained variance, was positively correlated with Age (*r* = 0.34, FDR < 0.10), Vaccine (*r* = 0.34, FDR < 0.10), and Timepoint (*r* = 0.44, FDR < 0.05). Two PCs, PC3 and PC4, accounting for 7.67 and 5.94% of total explained variance, respectively, demonstrated significant correlations with Timepoint but not Vaccine; PC3 demonstrated negative correlation with Timepoint (*r* = −0.38, FDR < 0.10) and ID (*r* = −0.39, FDR < 0.10) and PC4 demonstrated positive correlation with Timepoint (*r* = 0.38, FDR < 0.10) and Age (*r* = 0.47, FDR < 0.05), confounded by ID (*r* = −0.38, FDR < 0.10). Accounting for 5.74% of total explained variance, PC5 possessed significant negative correlation with Timepoint (*r* = −0.32, FDR < 0.10). While confounded by Pasture (*r* = −0.54, FDR < 0.01), PC10, accounting for 2.72% of total explained variance, possessed significant positive correlation with Vaccine (*r* = 0.36, FDR < 0.10). Notably, the strongest correlation found within this analysis was between PC11, accounting for 2.33% of total explained variance, and GR (*r* = 0.58, FDR < 0.01). The resulting pairwise plotting of PCs 1, 3, 4, 5, and 10 demonstrated relative overlapping of all samples at T1, with increasing dissimilarity of samples over time (Figure 3B). A biplot with statistical ellipses (multivariate C.I. = 80.00%) of the two PCs with significant correlation with Vaccine (PC1 and PC10) demonstrated high dissimilarity between timepoints T1 and T3, with relative high overlap of timepoints T2 and T4, with T3 variation driven by vaccinated calves J009, J022, J023, and J113 (Figure 3C). Genes driving the variation among each PC possessing significant metadata correlations are found in Figure 3E. Specifically, genes influencing variation within PC1 and PC10 (i.e., correlated PCs with Vaccination) include *AP5M1, CLOCK, EIF3K, HDAC3, MKLN1, MYNN, OCIAD1, PHIP, RACK1, RBM12B, RBM26, RPL37A, SNX17, STK16, TMEM208, TRAPPC1, UBXN7*, and *ZDHHC17* in PC1 and *KIR3DL1, LOC112447728*, and *LOC786987* in PC10, respectively. Those PCs having significant correlation with Timepoint, and not Vaccine (PC3, PC4, and PC5), possessed variance-driving genes which overlapped with glmmSeq–timepoint findings; *BATF, EXTL2, PRDX2, RNF122, TIAM1*, and *TMCC3* were identified in both PC3-5 loadings plots and glmmSeq–timepoint analysis.

**Figure 4:**
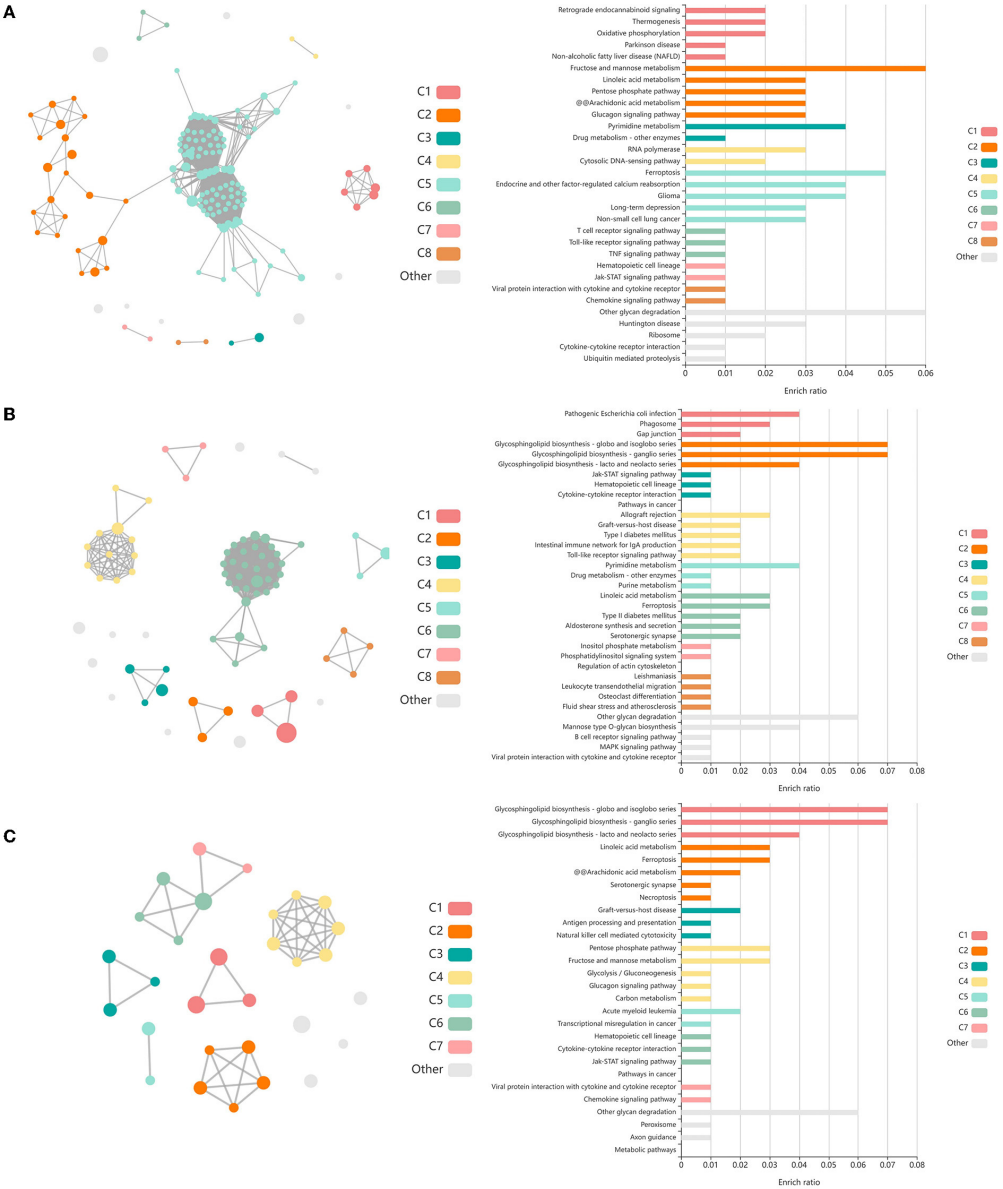
Clustering of enriched KEGG pathways by term identity from KOBAS-i analysis of DEGs influenced by time in both vaccination groups. Each node represents an enriched term, with color corresponding to the unique cluster based on term identity. Each edge (line between nodes) represents a significant correlation between pathway terms. Bar graphs represent the pathway terms found within each pathway (by color) and the level of enrichment (Enrich ratio). Gray nodes and bargraphed terms represent enriched pathways which did not associate within the clustering model. **(A)** KEGG pathways derived from T1 vs. T3 analysis clustered into eight unique clusters. **(B)** KEGG pathways derived from T1 vs. T4 analysis clustered into eight unique clusters. **(C)** KEGG pathways derived from T2 vs. T4 analysis clustered into seven unique clusters.

**Figure 5:**
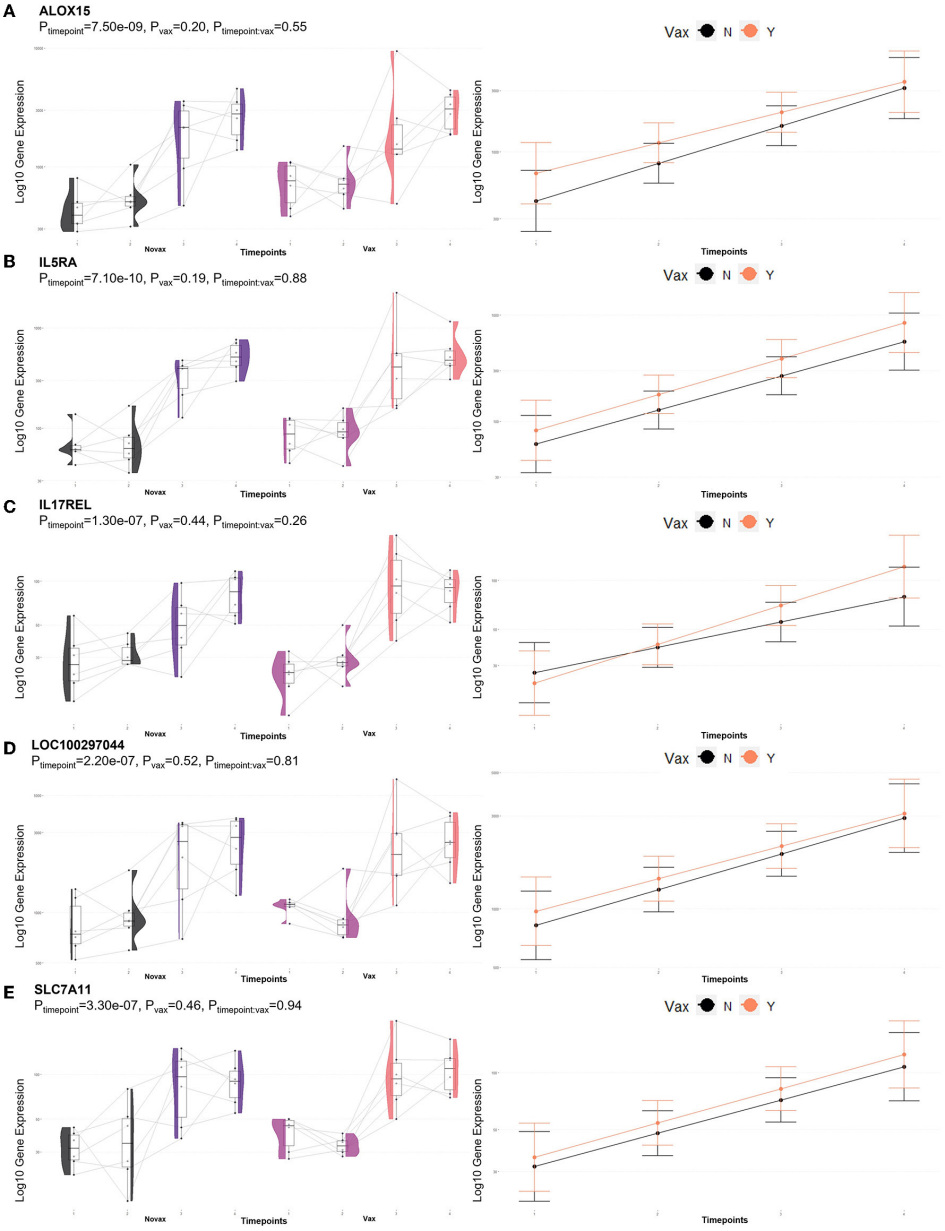
Gene pairplots and modeled expression trends of key DEGs found in timepoint analyses. Pairplots (left side) demonstrate the log10 normalized gene expression of each sample across all timepoints, overlapped with a violin plot (depicting numerical distributions by density). Box-and-whisker plots represent median expression values (black line), the first (lower) and third quartiles (boxplot limits), 1.5 times the interquartile ranges (whiskers), and outlier expression levels for each timepoint (points outside whiskers). Modeled expression trends (right side) depict the overall differences between groups over each timepoint. Points represent the mean log10 normalized expression value for each group within a timepoint, and bars represent the standard error of log10 normalized expression for each group; orange represents the vaccinated group and black represents the non-vaccinated group. These plots depict the relative expression and glmmSeq level of significance for **(A)** *ALOX15*, **(B)** *IL5RA*, **(C)** *IL17REL*, **(D)** *LOC100297044* (*CCL14*), and **(E)** *SLC7A11*.

Analysis of GO terms, KEGG pathways, and Reactome pathways of genes identified between glmmSeq–timepoints and edgeR QLF testing within both vaccination groups across time allowed for the assessment of enriched processes and pathways at three specific timepoint comparisons: (1) T1 vs. T3, (2) T1 vs. T4, and (3) T2 vs. T4 (Supplementary material 7). Shared DEGs from T1 vs. T3 comparisons enriched for 88 GO terms and 72 functional pathways. These GO terms were related to zinc ion binding, cytokine-mediated signaling, specifically interleukin-12, gene expression regulation, regulation to inflammatory response, including negative regulation of I-kappaB kinase/NF-kappaB signaling, and fatty acid metabolism and biosynthesis. Enriched pathways included the immune system (both innate and acquired immunity) retrograde endocannabinoid signaling, interleukin-4/13 signaling, glucose metabolism, glucagon signaling, TP53 expressional and degradation regulation, and the biosynthesis of specialized proresolving mediators (SPMs), including SPMs derived from both docosahexaenoic acid (DHA) and eicosapentaenoic acid (EPA). These GO terms and pathways were primarily enriched by the following DEGs: *ADAMTS12, ALOX15, ALOX5, CFL1, CPT1A, FBP1, FSCN1, IL5RA, LOC100297044* (*CCL14*), *LOC615278* (*TRIM39*), *LOC789732* (*CD300C*), *MIF, OTUD7B, PEG10, PIKFYVE, PLP2, POLR2L, PPP2R1A, PRKCG, PYGM, TK1*, and *TP53*. Shared DEGs from T1 vs. T4 comparisons enriched for 34 GO terms and 35 functional pathways. These GO terms were related to inflammatory response, cytokine-mediated signaling, magnesium ion binding, cellular response to oxidative stress, positive regulation of autophagy, T-cell co-stimulation, and actin/microtubule organization and development. Enriched pathways included the acquired immune system, interleukin signaling, cellular stress response, CD28 co-stimulation and signaling, and gap junction trafficking and regulation. These GO terms and pathways were primarily enriched by the following DEGs: *ALOX15, CD80, HMGA1, HSPB8, IL17REL, IL5RA, LOC100297044* (*CCL14*), *LOC533307* (*LRRK2*), *LOC789732* (*CD300LD*), *MAP3K8, NCF2, SLC7A11, TUBB, TUBB3*, and *ZC3H12A*. Shared DEGs from T2 vs. T4 comparisons enriched for 94 GO terms and 29 functional pathways. These GO terms were related to inflammatory and cytokine-mediated response, specifically including interleukin-17 receptor activity, MHC class I protein complex binding, response to mercury and magnesium ions, antigenic stimuli and macrophage differentiation, and fatty acid metabolism and biosynthesis. Enriched pathways included cellular metabolism involving fructose, mannose, pyruvate, and lipid metabolism, cytokine-cytokine receptor interaction, and the biosynthesis of specialized proresolving mediators (SPMs), including SPMs derived from both docosahexaenoic acid (DHA) and eicosapentaenoic acid (EPA). These GO terms and pathways were primarily enriched by the following DEGs: *ALOX15, CEBPE, DECR2, FBP1, IL17REL, IL5RA, LOC100297044* (*CCL14*), *LOC788694* (*KLRC1*), and *SLC7A11*. Visualization of the enriched KEGG pathway terms is found in Figure 4. Expressional trends of DEGs identified in all three timepoint comparisons between the two vaccination groups (*ALOX15, IL5RA, IL17REL, LOC100297044* (*CCL14*), and *SCL7A11*) are found in Figure 5.

**Figure 6:**
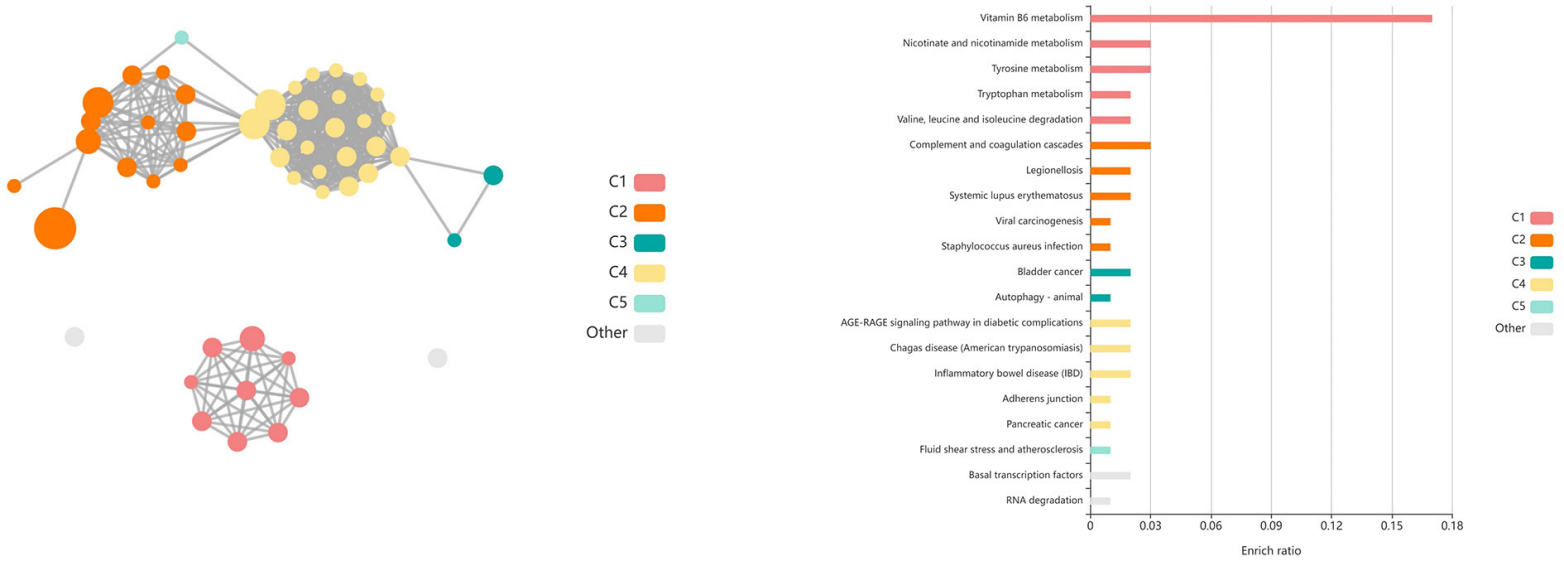
Clustering of enriched KEGG pathways by term identity from KOBAS-i analysis of DEGs identified between vaccinated and non-vaccinated calves at T2. Each node represents an enriched term, with color corresponding to the unique cluster based on term identity. Each edge (line between nodes) represents a significant correlation between pathway terms. Bar graphs represent the pathway terms found within each pathway (by color) and the level of enrichment (Enrich ratio). KEGG pathways identified between vaccine groups at T2 clustered into five unique clusters. Gray nodes and bar graphed terms represent enriched pathways which did not associate within the clustering model.

**Figure 7:**
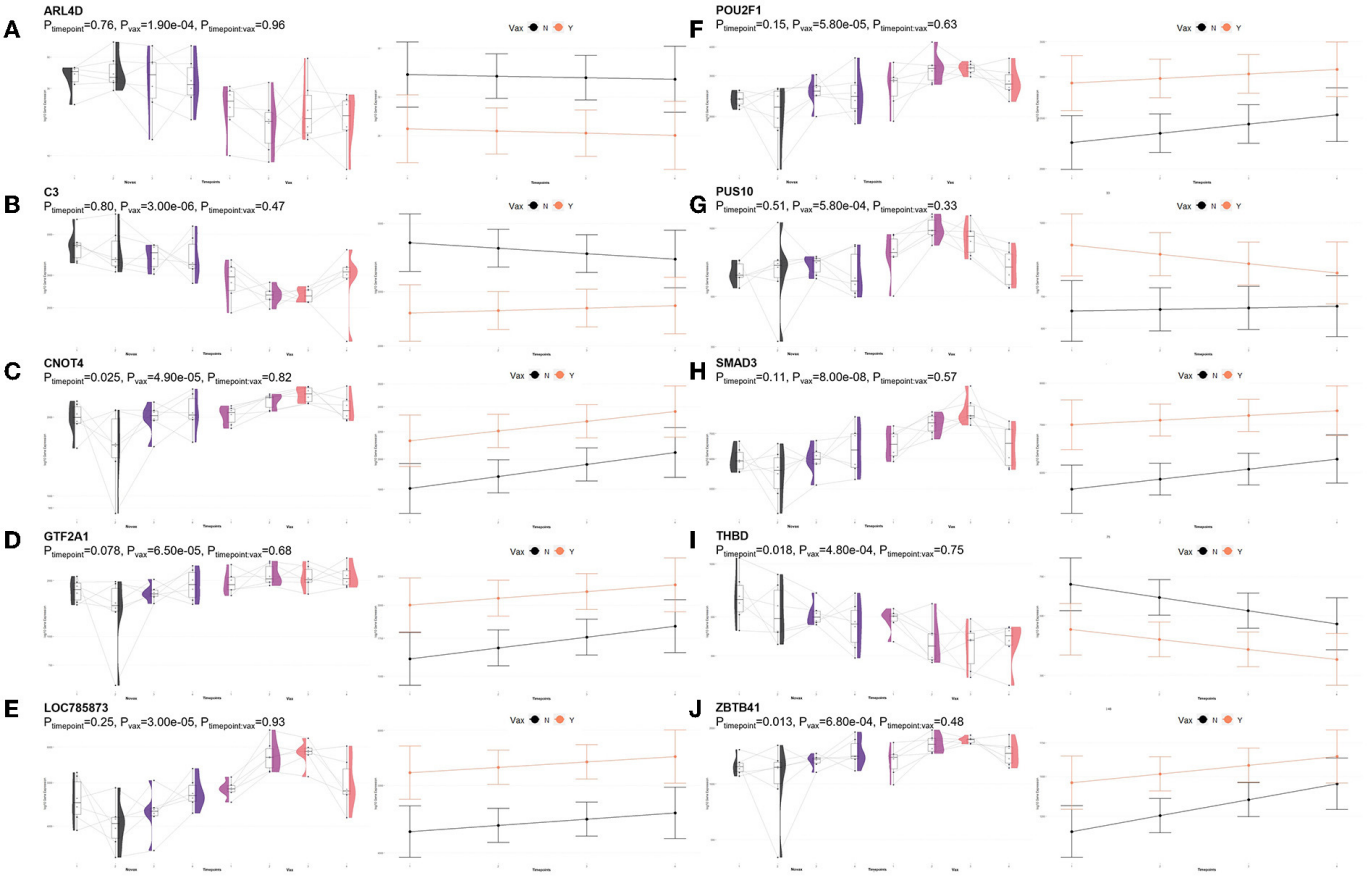
Gene pairplots and modeled expression trends of key DEGs found in timepoint analyses. Pairplots (left side) demonstrate the log10 normalized gene expression of each sample across all timepoints, overlapped with a violin plot (depicting numerical distributions by density). Box-and-whisker plots represent median expression values (black line), the first (lower) and third quartiles (boxplot limits), 1.5 times the interquartile ranges (whiskers), and outlier expression levels for each timepoint (points outside whiskers). Modeled expression trends (right side) depict the overall differences between groups over each timepoint. Points represent the mean log10 normalized expression value for each group within a timepoint, and bars represent the standard error of log10 normalized expression for each group; orange represents the vaccinated group and black represents the non-vaccinated group. These plots depict the relative expression and glmmSeq level of significance for **(A)** *ARL4D*, **(B)** *C3*, **(C)** *CNOT4*, **(D)** *GTF2A1*, **(E)** *LOC785873 (TRIM26)*, **(F)** *POU2F1*, **(G)** *PUS10*, **(H)** *SMAD3*, **(I)** *THBD*, and **(J)** *ZBTB41*.

A total of 435 genes were identified by glmmSeq-Vaccination to be differentially expressed (Supplementary material 3), with 109 unique DEGs identified by overlapping glmmSeq–Vaccination and GLM-QLF testing results, post-removal of DEGs identified in Timepoint evaluation (Supplementary material 8). Specifically, a total of one, 24, and 92 DEGs were identified between vaccination groups at timepoints T1, T2, and T3, respectively; no genes were found to be differentially expressed between vaccinated and non-vaccinated calves at T4 (Supplementary material 8). Only one DEG was identified at T1 (*HEXDC*; increased in Vaccinated) between vaccinated and non-vaccinated calves, therefore possessed no enriched GO terms nor pathways. Shared DEGs identified at T2 between vaccinated and non-vaccinated calves enriched for 139 GO terms and 61 functional pathways. These GO terms were related to immune response and regulation (increased in Vaccinated), T-cell activation (increased in Vaccinated), metal ion binding (increased in Vaccinated), positive transcriptional regulation and protein processing (increased in Vaccinated), cellular proliferation and maintenance (increased in Vaccinated), complement activity (decreased in Vaccinated), and apoptotic clearance and phagocytosis (decreased in Vaccinated). Enriched pathways included the immune system and cytokine signaling, including interleukin-37 signaling (increased in Vaccinated), complement and coagulation cascades (decreased in Vaccinated), enhanced transcriptional activity, largely involving RNA polymerase II (increased in Vaccinated), and vitamin B6 metabolism (decreased in Vaccinated). These GO terms and pathways were primarily enriched by the following DEGs: *ARL4D, C3, CNOT4, GTF2A1, LOC785873* (*TRIM26*), *POU2F1, PUS10, SMAD3, THBD*, and *ZBTB41*. Visualization of the enriched KEGG pathways is found in Figure 6. Expressional trends of the aforementioned DEGs contributing to these GO terms and pathways are found in Figure 7.

**Figure 8:**
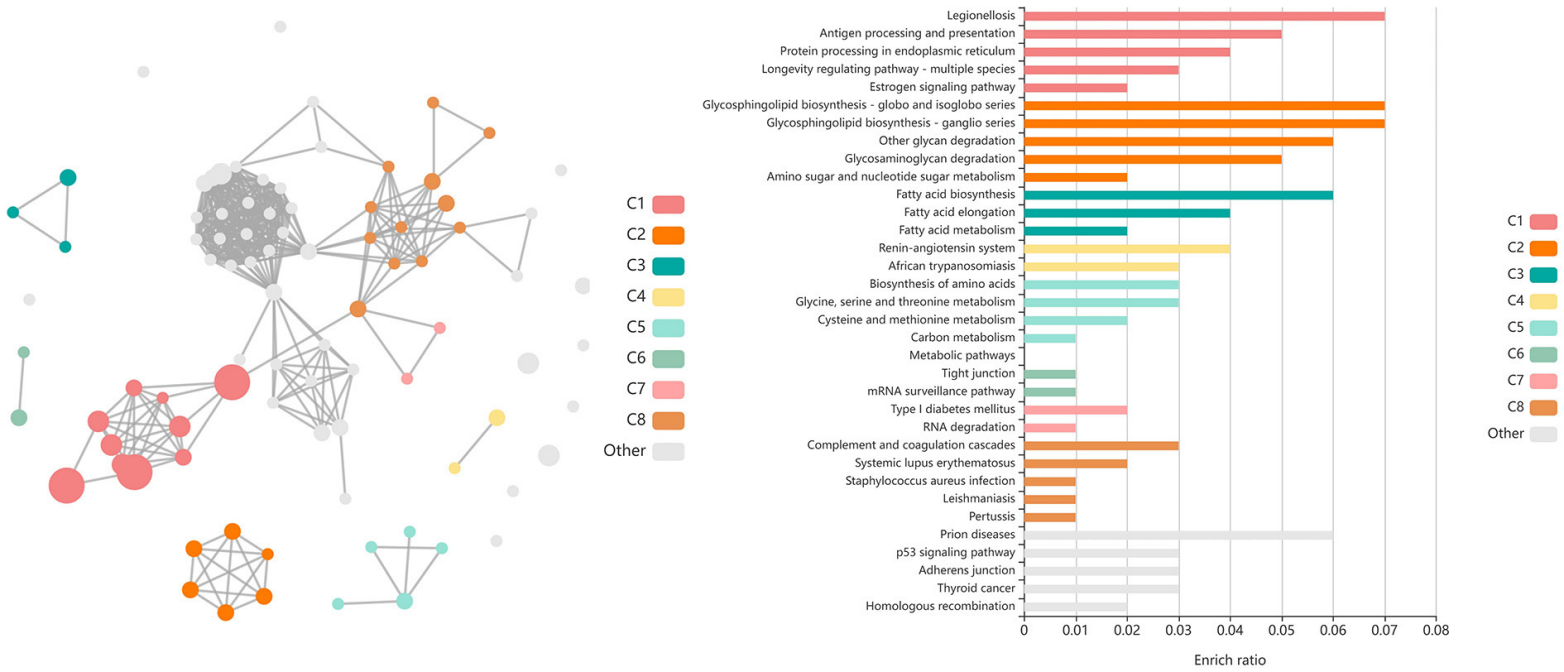
Clustering of enriched KEGG pathways by term identity from KOBAS-i analysis of DEGs identified between vaccinated and non-vaccinated calves at T3. Each node represents an enriched term, with color corresponding to the unique cluster based on term identity. Each edge (line between nodes) represents a significant correlation between pathway terms. Bar graphs represent the pathway terms found within each pathway (by color) and the level of enrichment (Enrich ratio). KEGG pathways identified between vaccine groups at T3 clustered into eight unique clusters. Gray nodes and bar graphed terms represent enriched pathways which did not associate within the clustering model.

**Figure 9:**
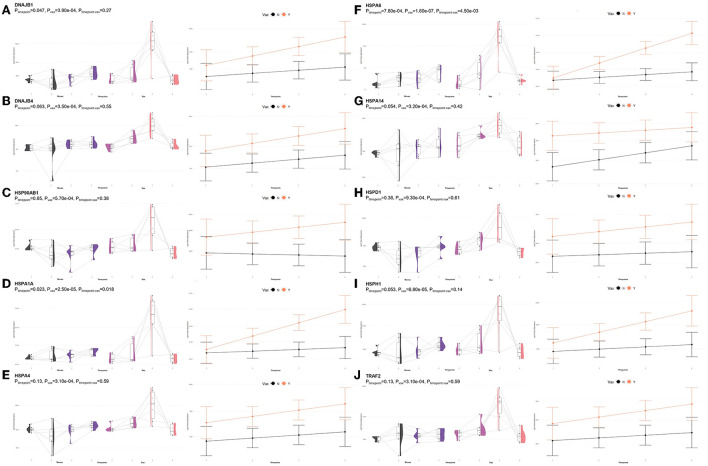
Gene pairplots and modeled expression trends of key DEGs found in timepoint analyses. Pairplots (left side) demonstrate the log10 normalized gene expression of each sample across all timepoints, overlapped with a violin plot (depicting numerical distributions by density). Box-and-whisker plots represent median expression values (black line), the first (lower) and third quartiles (boxplot limits), 1.5 times the interquartile ranges (whiskers), and outlier expression levels for each timepoint (points outside whiskers). Modeled expression trends (right side) depict the overall differences between groups over each timepoint. Points represent the mean log10 normalized expression value for each group within a timepoint, and bars represent the standard error of log10 normalized expression for each group; orange represents the vaccinated group and black represents the non-vaccinated group. These plots depict the relative expression and glmmSeq level of significance for **(A)** *DNAJB1*, **(B)** *DNAJB4*, **(C)** *HSP90AB1*, **(D)** *HSPA1A*, **(E)** *HSPA4*, **(F)** *HSPA6*, **(G)** *HSPA14*, **(H)** *HSPD1*, **(I)** *HSPH1*, and **(J)** *TRAF2*.

Shared DEGs identified at T3 between vaccinated and non-vaccinated calves enriched for 71 GO terms and 25 functional pathways. These GO terms were related to neutrophil degranulation (increased in Vaccinated), antigen processing and presentation (increased in Vaccinated), ubiquitin protein binding and positive regulation (increased in Vaccinated), nuclear protein importing and response to protein folding (increased in Vaccinated), heat shock protein binding, specifically to Hsp70 and Hsp90 (increased in Vaccinated), T-cell activation (increased in Vaccinated), cellular response to interleukin-7 (increased in Vaccinated), and the positive regulation to ATPase activity (increased in Vaccinated). Enriched pathways included transcription activation (increased in Vaccinated), endocytosis and antigen processing and presentation (increased in Vaccinated), neutrophil degranulation (increased in Vaccinated), and cellular response to heat stress, including the regulation of HSF1-mediated heat shock response, Hsp90 chaperone cycle for steroid hormone receptors, and HSF1-dependent transactivation (all increased in Vaccinated). These GO terms and pathways were primarily enriched by the following DEGs: *AHSA2, BANP, C3, CACYBP, CCT2, DNAJA4, DNAJB1, DNAJB4, HIST1H3G, HSP90AB1, HSPA14, HSPA1A, HSPA4, HSPA6, HSPD1, HSPH1, KAT2A, MDM4, NUTF2, PTPRB, RAB11FIP3, RCHY1, SMAD3, STIP1, SYMPK, TOMM34, TRAF2, ZFAND2A, ZFP28*, and *ZNF473*. Visualization of the enriched KEGG pathway terms is found in Figure 8. Expressional trends of the DEGs primarily involved in immune mediated and heat shock response associated GO terms and pathways (*DNAJB1, DNAJB4, HSP90AB1, HSPA14, HSPA1A, HSPA4, HSPA6, HSPD1, HSPH1*, and *TRAF2*) are found in Figure 9.

**Figure 10:**
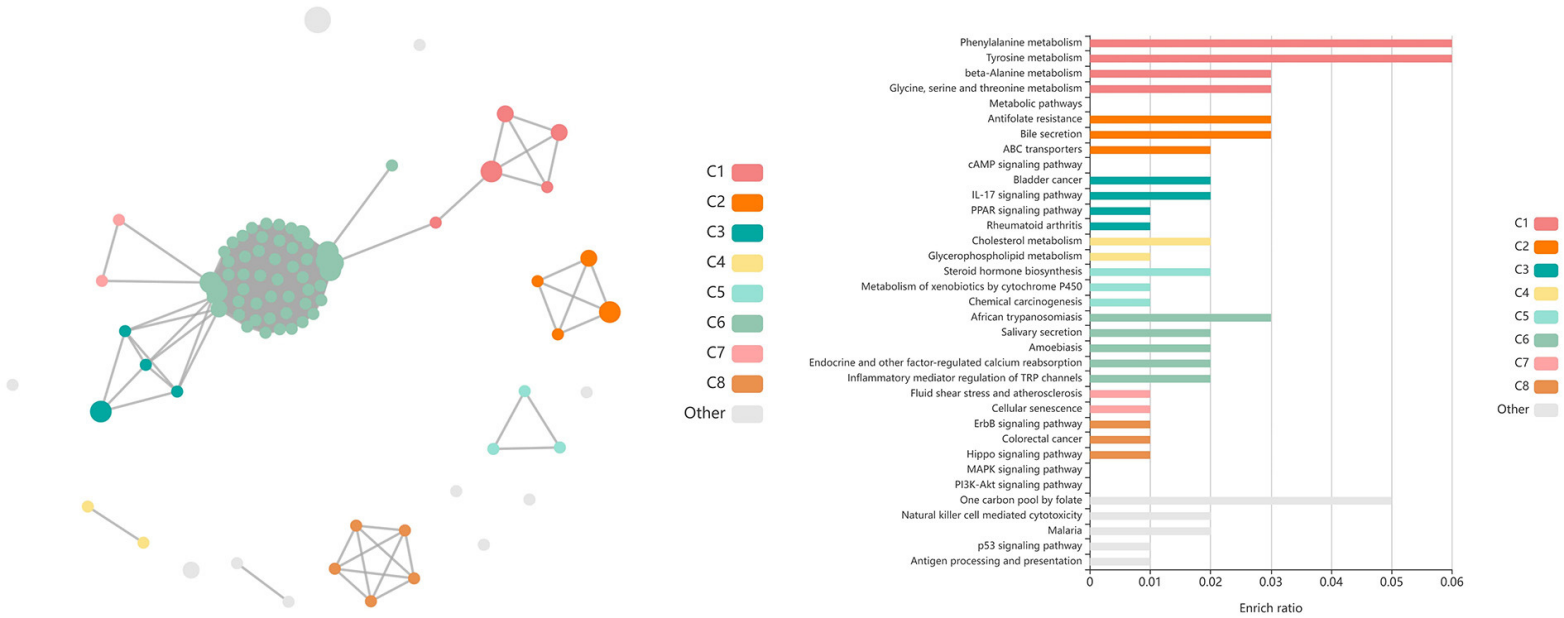
Clustering of enriched KEGG pathways by term identity from KOBAS-i analysis of DEGs identified from glmmSeq evaluation of the interaction between vaccination and time. Each node represents an enriched term, with color corresponding to the unique cluster based on term identity. Each edge (line between nodes) represents a significant correlation between pathway terms. Bar graphs represent the pathway terms found within each pathway (by color) and the level of enrichment (Enrich ratio). KEGG pathways identified through the interaction of vaccination and time clustered into eight unique clusters. Gray nodes and bar graphed terms represent enriched pathways which did not associate within the clustering model.

**Figure 11:**
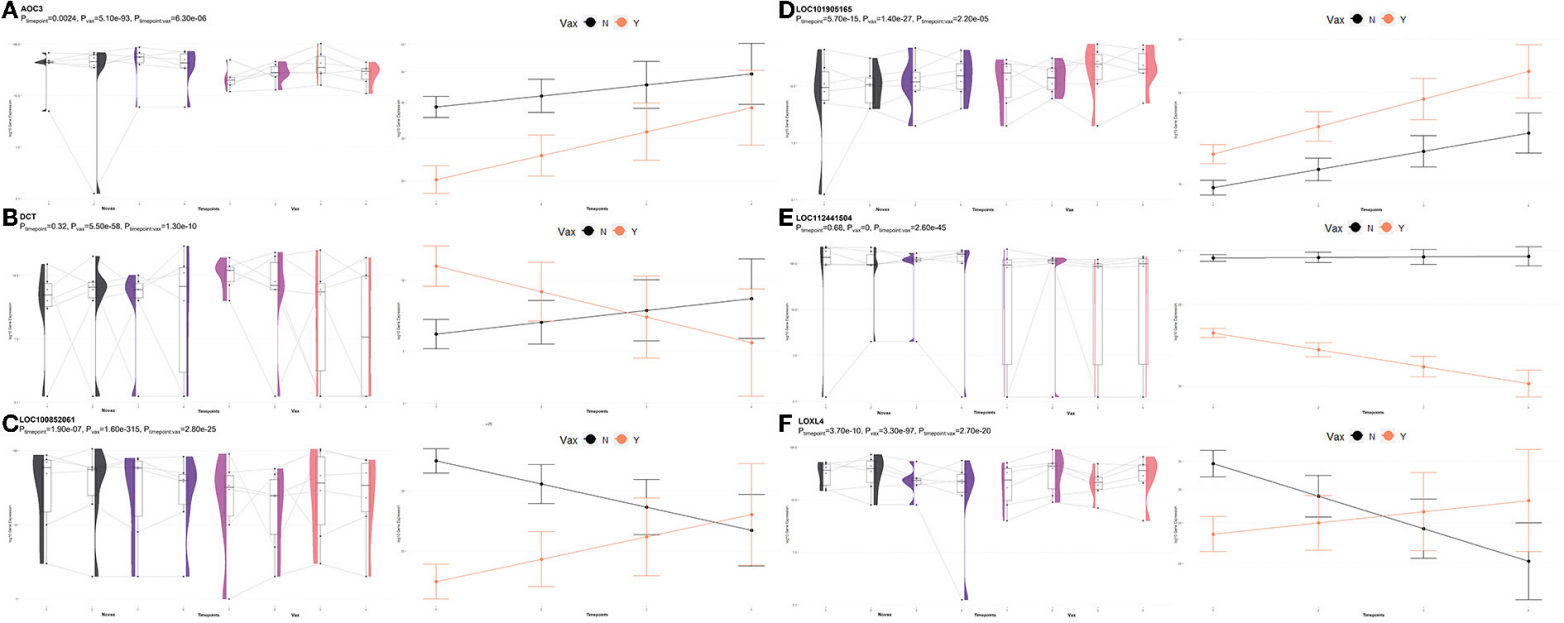
Gene pairplots and modeled expression trends of key DEGs found in timepoint analyses. Pairplots (left side) demonstrate the log10 normalized gene expression of each sample across all timepoints, overlapped with a violin plot (depicting numerical distributions by density). Box-and-whisker plots represent median expression values (black line), the first (lower) and third quartiles (boxplot limits), 1.5 times the interquartile ranges (whiskers), and outlier expression levels for each timepoint (points outside whiskers). Modeled expression trends (right side) depict the overall differences between groups over each timepoint. Points represent the mean log10 normalized expression value for each group within a timepoint, and bars represent the standard error of log10 normalized expression for each group; orange represents the vaccinated group and black represents the non-vaccinated group. These plots depict the relative expression and glmmSeq level of significance for **(A)** *AOC3*, **(B)** *DCT*, **(C)**, *LOC100852061* (*KIR2DS2*), **(D)** *LOC101905165* (*NKG2D*), **(E)** *LOC112441504* (*ULBP3*), and **(F)** *LOXL4*.

A total of 85 DEGs were identified by glmmSeq when evaluating the interaction between Vaccination and Timepoints, which enriched for 13 GO terms and six functional pathways (Supplementary material 9). These GO terms were related to extracellular space, actin filament organization, cytoplasmic vesicles, copper ion binding, and natural killer cell activation and mediated cytotoxicity. Enriched pathways included small molecule transport, immunoregulatory interactions between lymphoid and non-lymphoid cells, plasma lipoprotein remodeling, natural killer cell mediated cytotoxicity, tyrosine metabolism, and DAP12 interactions. Visualization of the enriched KEGG pathway terms is found in Figure 10. Expressional trends of the DEGs primarily involved in immunoregulatory and natural killer cell associated GO terms and pathways (*AOC3, DCT, LOC100852061* (*KIR2DS2*), *LOC101905165* (*NKG2D*), *LOC112441504* (*ULBP3*), and *LOXL4*) is found in Figure 11.

## Discussion

### Use of modified live viral respiratory vaccines in beef cattle production systems

The use of modified live viral (MLV) vaccines in beef cattle backgrounding and feeding operations remains one of the leading practices in managing risk of BRD in cattle populations (1). Multiple recent reviews have evaluated the peer-reviewed literature regarding the use of various vaccines for respiratory pathogens in beef cattle (20–22). However, vaccination is not always helpful (23) and questions remain regarding which cattle are most likely to benefit from vaccination and which may not. Assessment of the transcriptome may reveal new pathways that will explain why vaccination appears to prevent disease in certain situations but not others. The significance of some of the differences in observed gene expression between VAX and NOVAX calves is not yet clear, but provides a foundation for future studies to determine how multiple components of the immune response change following vaccination. To our knowledge, there is no comparable data set available.

Although variable in terms of individual efficacy, several studies suggest that vaccinating herds of cattle with MLV vaccines reduces herd-level risk of BRD-associated morbidity and mortality and is associated with improved weight gain overtime (i.e., production) (24–27). Our study was limited to a small subset of calves that remained clinically healthy which may have influenced the subsequent lack of difference in performance.

For years, responses to vaccination have been measured *via* serology (4), and occasionally, cell-mediated immune responses (20). Such studies usually describe only a small number of outcomes of a vast and diverse network of interactions that influence health vs. disease. While we attempted to assess serum neutralizing titer responses to the viruses we vaccinated against, the timing of sample collection was not optimized to find peak titer responses. Additionally, it is important to note that in this study the MLV was administered differently than the label directions indicated. The current label for Pyramid 5 (28) does not indicate a minimum age requirement or a specific interval or requirement for revaccination of calves. However, administering a booster vaccination, especially when the primary vaccination was given to animals under 6 months of age, is common industry practice and according to current knowledge would be helpful in initiating a protective immune response. Our serology results indicated that the calves responded to our vaccination strategy as expected but that there was likely a natural exposure to PI-3 and BRSV in the herd. The lack of differential gene expression at T4 between VAX and NOVAX calves is further supported by the titer data at T4. This may be due to the length of time between sampling points T3 and T4, but our data suggest both VAX and NOVAX individuals, across multiple pens, were exposed to a potentially non-virulent strain of PI-3 and BRSV sometime between T3 and T4. Furthermore, the similarities in gene expression between the two groups at T4 may be confounded due to this exposure and processing at T3. However, the results of this study demonstrated that the driver of immunological response and enhanced transcription over time, as influenced by vaccination, was the initial (first) administration.

### Development of clinically healthy cattle is associated with increased specialized proresolving mediator expression, fatty acid and carbohydrate metabolism, and cytokine-mediated immunity

When evaluating the influence of time (i.e., physiological growth) on the gene expression of young calves, three connected mechanisms continually increased over time across all individuals: specialized proresolving mediator (SPM) biosynthesis, fatty acid and carbohydrate metabolism, and chemokine/cytokine mediated enhancement of acquired immunity. Specialized proresolving mediators consist of closely related classes of lipid mediators, derived from the lipoxygenation of arachidonic acid into LXA4 (i.e., lipoxins) (29) or from the metabolism of omega-3 and/or omega-6 essential polyunsaturated fatty acids (i.e., resolvins, protectins, and maresins) (30–32). Collectively, six molecules (*ALOX5, ALOX15, GPX4, HPGD, LTA4H*, and *PTGS2*) are directly involved in the biosynthesis of SPMs,^5^ of which we identified three to be differentially increased in all calves over time (*ALOX5, ALOX15*, and *HPGD*); notably, *HPGD* was identified as a differentially expressed in glmmSeq – timepoint, NOVAX T1vT4, VAX T1vT3, and VAX T2vT3. These lipid molecules are profound regulators of both acute and chronic inflammation and are critical in promoting cellular clearance and tissue remodeling in response to respiratory disease (33–36). Recent evidence suggests that, in addition to their ability to resolve inflammatory responses and tissue damage, SPMs are effective modulators of the adaptive immune response, capable of regulating Th1/Th17 differentiation and promoting regulatory T-cell differentiation *via* a non-cytopathic regulatory mechanism (37). Crucially, SPMs are shown to not have an effect on Th2-driven immunity, but enhance antigen presenting cell, specifically dendritic cell, development and functionality (37–39); this aligns with our findings indicating a gradual increase in gene expression related to SPM production and immunoregulatory T-cells. This is additionally supported by the enrichment of CD28 co-stimulation and signaling, and the enhancement of *CTLA4* and *CD80* with associated cytokine production (*IL5RA, IL17REL*) over time (40–44). Furthermore, several of these specific genes, namely *ALOX15, LOC100297044* (CCL14), *HPGD*, and *IL5RA*, have been identified as DEGs increased in expression at facility arrival in cattle that remain clinical healthy within high-risk populations, compared to cattle that develop BRD (45–49). Collectively, this may represent immunological development and mechanisms of immunocompetence which can serve a protective role against BRD-induced inflammation when calves are placed in post-weaned feeding systems.

### Vaccination induces a controlled inflammatory response linked with Th17/natural killer cell activity

Evaluation of host expression influenced by vaccination, excluding genes and mechanisms affected solely by time, demonstrated an increase in mechanisms associated with antigen presentation, metal ion binding, molecular chaperone activity, and lymphoid cell activity, and a decrease in mechanisms associated with complement and apoptotic debris clearance. First, through PCA of global expression trends, we discovered genes driving variation in PCs with significant correlation with vaccination. Specifically, we identified the genes *CLOCK, HDAC3, KIR3DL1, RACK1*, and *SNX17* to be key drivers of differences associated with vaccination, which are involved in regulating the activity and differentiation of T-cells and natural killer cells. *CLOCK*, in conjunction with *BMAL1*, is a circadian timekeeping protein which interacts with transcriptional regulators, which in turn upregulate genes such as *HDAC3*; this transcriptional network is responsible for the development and differentiation of Th17 cells (50–53). *KIR3DL1*, an immunoglobulin-like receptor expressed by natural killer cells and T-cells (54), is shown to be involved in inhibiting interferon-?? secretion and may block the progression of chronic inflammation, seen in research involving ankylosing spondylitis and reactive arthritis (55, 56). *RACK1*, which acts as both an intracellular protein receptor for protein kinase C and as a core ribosomal protein of the 40S subunit, is a key component of T-cell activation and proliferation (57, 58) and loss of *RACK1* has been shown to increase T-cell apoptosis (59). *SNX17* localizes with T-cell receptors and is responsible for preventing T-cell degradation into lysosomes and transporting T-cell receptors to the cell surface, aiding in cellular immune function (60). These findings provide initial evidence that vaccination in young calves influences mechanisms related to the enhanced differentiation and survival of T-cells, natural killer cell activity, and accompanying interleukin-17 response; this coincides with previous research demonstrating vaccination or exposure to viral components mediates a T-helper cell and natural killer cell response (61, 62), which may contribute to protective cell mediated and controlled inflammatory responses (63).

To further explore the influence of vaccination on these calves, we identified DEGs between vaccination groups at each time point, and those found from the interaction between vaccination and time. At T2 (7 days post vaccination), DEGs identified in calves which received a MLV vaccination enriched for two major immune-related mechanisms-the downregulation of complement and coagulation cascades (primarily driven by *C3*), and the upregulation of T-cell-mediated immunity. Complement, a well-organized and highly regulated system of the immune system, is a critical component of host immunity for killing or neutralizing pathogens and maintaining immunological homeostasis (64, 65). While the complement system features three distinct response pathways (classical, alternative, and mannose-binding lectin), all three lead to subsequent *C3* activation (66). Interestingly, the vaccinated group demonstrated a downregulation of *C3* transcription. While complement *C3* is critical for inducing a humoral and cell-mediated response to vaccination and viral infection (67–70), little published information exists relating to the timing and activity levels of induced complement cascades in cattle. Thus, it can be hypothesized that we failed to capture the initial immune responses associated with vaccination within the first few days and are identifying a late feedback mechanism involved in controlling prolonged complement activity. Additionally, research has demonstrated that the complement system appears to be more important for successful immunization in response to polysaccharide-containing vaccines compared to conjugated vaccines (71). Furthermore, we identified DEGs and enriched mechanisms related to CD4+ T-cell activity, primarily driven by *DAPK2, POU2F1, SMAD3*, and *LOC785873* (*TRIM26*). *DAPK2* promotes cellular recruitment to sites of inflammation (72) and is highly expressed in activated T-cells, serving a cellular regulatory role during germinal center formation (73). *POU2F1* is a required transcription factor for T-cell response to infection and the development of CD4+ memory T-cells (74–76). *SMAD3* transduces TGF-BR signaling and controls the development of regulatory T-cells and Th17 cells v*ia* signaling networks involving T-cell receptors, TGF-B, and interleukin-6 (77, 78). While not directly involved with T-cell activity, *TRIM26* is involved with modulating host antiviral defense and inducing an inflammatory immune response (79–81).

Evaluation of T3 (prior to booster administration; 77 days after initial vaccination) identified DEGs and enriched immunological mechanisms involved in neutrophil degranulation, antigen processing and interleukin-7 response, T-cell activation, transcriptional activity, and heat shock protein activity and binding. At this time point in vaccinated calves compared to non-vaccinated calves, we again observed an increase in the expression of *SMAD3* and *LOC785873* (*TRIM26*), two genes involved in T-cell development and inflammatory defense mechanisms, respectively, and a decrease in *THBD* and *C3*, involved in coagulation (82, 83) and complement activity, respectively. Surprisingly, these genes and associated mechanisms remained significantly enriched at both seven and 77 days post-vaccinated, indicating a possible immune-mediated mechanism or complex induced by MLV vaccination which persists longer than anticipated (>30 days). One unexpected finding at this timepoint was the rapid increase in heat shock protein gene expression in vaccinated calves. There is a great deal of research demonstrating the role of heat shock proteins in vaccination and host immunity/inflammation. Hsp70 enhances immunogenic antigen presentation cell functionality and T-cell proliferation (84). Both Hsp70 and Hsp90 proteins are shown to activate dendritic cells and direct naïve helper T-cell priming through designated interactions with antigen presenting cell surface receptors (85, 86) and stimulating inflammatory cytokine production *via* CD14-mediated chaperoning (87–89). *HSPD1* initiates interferon-beta production through interactions with interferon regulatory factor 3 (*IRF3*) (90) and is associated with both leukocyte and lymphocyte tissue infiltration (91). Furthermore, both Hsp70 and Hsp90 promote Th17 gene expression and proliferation and are involved in interleukin-17-mediated inflammation (92–94). This collectively indicates a stimulation of heat shock protein-mediated inflammation and helper T-cell, possibly Th17, promotion *via* modified live viral vaccination. However, this mechanism was only upregulated at T3. How long and where in time this mechanism becomes upregulated through viral vaccination could not be fully elucidated by this study and additional research is needed.

Our final differential expression evaluation was to determine genes influenced by the interaction of both time and vaccination. Largely, the DEGs identified through this analysis were determined to be involved with immunoregulatory functions *via* natural killer cells. These mechanisms were enriched by three key genes: *LOC101905165* (*NKGD2*), *LOC100852061* (*KIR2DS2*), and *LOC112441504* (*ULBP3*). *NKG2D* serves as a costimulatory transmembrane receptor on natural killer cells, enhancing T-cell receptor activity and subsequent cytotoxic and gamma-delta T-cell function (95–97). *KIR2DS2* is an activating receptor of natural killer cells, which binds to MHC class 1 and enhances natural killer cell-mediated cytotoxicity (98, 99). *ULBP3* is a cellular ligand of natural killer cells which binds to *NKG2D*, serving an immunostimulatory role (100–102). This indicates that the influence of both time and vaccination acts in influencing natural killer cell and cytotoxic responses in calves. Bassi et al. (103) demonstrated that cattle naturally infected with and displaying clinical signs of bovine papillomavirus possessed an increase in circulating natural killer cells and CD4+/CD8+ ratios, with a related elevation in interleukin-17 levels, when compared to cattle without clinical papillomatosis. Hamilton et al. (104) found that BCG vaccination in neonatal calves induces effector natural killer cells after interactions with dendritic cells, and stimulates their production of type-2 interferon production and interleukin-12.

Another key detail in this study is the lack of differential expression related to type-1 interferon production and response. Research has demonstrated that administration of recombinant and mRNA vaccines against viral pathogens can induce type-1 interferon production, enhancing T-cell response *via* heighted antigen presentation, and further promoting humoral immunity and vaccine-induced antibody production (105–108). Previous research in cattle has demonstrated that type-1 interferon production is strongly induced by viral challenge and is seen as an antiviral defense mechanism (109–112). While type-1 interferon production in human vaccination trials is well documented, it is relatively unknown if ungulates possess a similar immune response. We may have failed to recognize such a response due to the time between sampling points. Further studies assessing additional time points post-vaccination and booster, and focused assessment of peripheral immune cell types and responses, are warranted to better identify and understand the complex interaction of mechanisms related to successful immunization.

This study is, to our knowledge, the first of its kind to describe differential gene expression pathways in calves over the first 7 months of life, and in relation to a commonly used vaccination scheme, in a longitudinal fashion. Our findings indicate that vaccination induces a controlled inflammatory response associated with natural killer cell and, likely, Th17 cell promotion. This is most likely a normal process of antigen presentation and immunological memory within calves, but still constitutes an inflammatory-inducing process. It may be hypothesized that these induced mechanisms are not effective when calves are placed in high-risk settings, where stress and inflammation are occurring, compared to the low-risk system in which these calves were studied.

## Data availability statement

The data presented in the study are deposited in the National Center for Biotechnology Information Gene Expression Omnibus (NCBI-GEO), accession number GSE205004.

## Ethics statement

The animal study was reviewed and approved by Mississippi State University Animal Care and Use Committee (IACUC protocol #19-169).

## Author contributions

SC, AW, and BK conceived and designed the vaccination study funded by USDA. MS, AW, BK, and KH managed the live animal project and collected samples and metadata for analysis. MS and SC coordinated diagnostics, analyzed the data, and wrote the manuscript. SC, MS, AW, BK, and KH edited the manuscript and approved the final version. All authors agree to be accountable for the content of the work.

## Funding

This project was supported by Agriculture and Food Research Initiative Competitive Grant no. 2019-67015-29845 from the USDA National Institute of Food and Agriculture. Additionally, this project was supported in part by internal funds provided by Texas A&M AgriLife Research and Texas A&M University School of Veterinary Medicine and Biomedical Sciences.

## Conflict of interest

The authors declare that the research was conducted in the absence of any commercial or financial relationships that could be construed as a potential conflict of interest.

## Publisher's note

All claims expressed in this article are solely those of the authors and do not necessarily represent those of their affiliated organizations, or those of the publisher, the editors and the reviewers. Any product that may be evaluated in this article, or claim that may be made by its manufacturer, is not guaranteed or endorsed by the publisher.

## Author disclaimer

Any opinions, findings, conclusions, or recommendations expressed in this publication are those of the author(s) and do not necessarily reflect the view of the U.S. Department of Agriculture nor the internal supporters of this project.

## Footnotes

^1^ https://www.bioinformatics.babraham.ac.uk/projects/fastqc/

^2^ https://github.com/KatrionaGoldmann/glmmSeq

^3^ https://CRAN.R-project.org/package=pheatmap

^4^ https://github.com/kevinblighe/PCAtools

^5^ https://reactome.org/PathwayBrowser/#/R-HSA-9018679&DTAB=MT
