# Supplementary figures and images for: Impact of preweaning vaccination on host gene expression and antibody titers in healthy beef calves
Source: Front Vet Sci. 2022 Sep 26;9:1010039. doi: 10.3389/fvets.2022.1010039 (PMC9549141; doi:10.3389/fvets.2022.1010039)

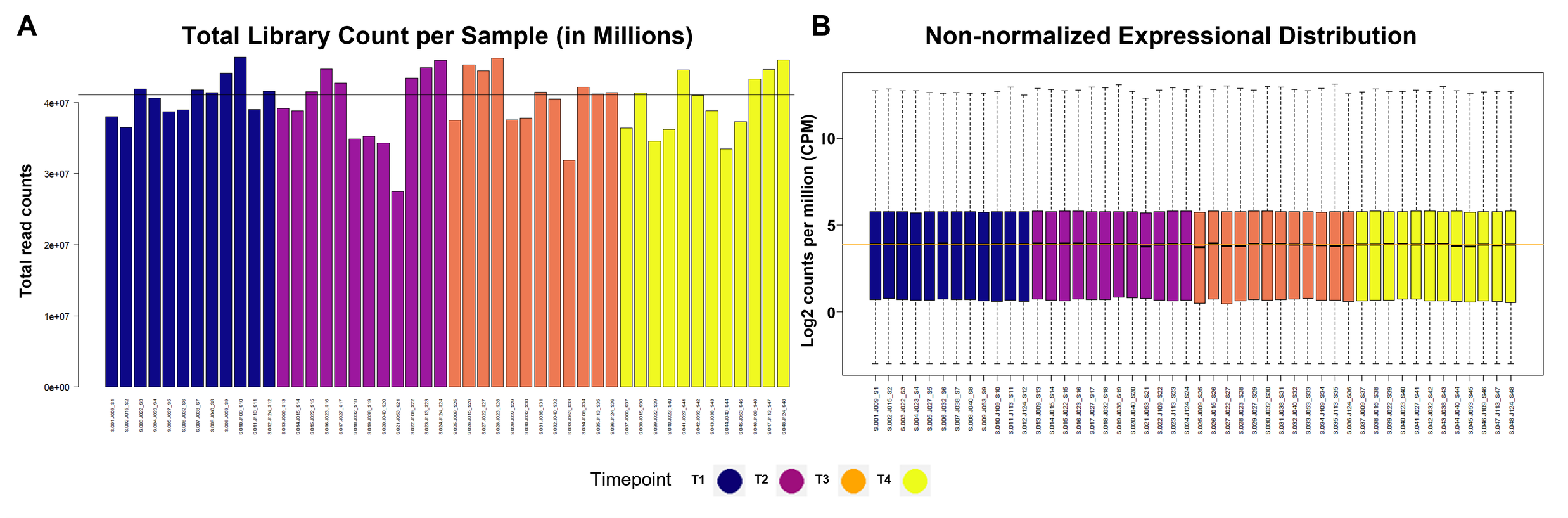

Supplement: Supplementary file 10 [file Image_1.TIF]
